# Barriers to global engagement for African researchers: A position paper from the Alliance for Medical Research in Africa (AMedRA)

**DOI:** 10.7189/jogh.14.03042

**Published:** 2024-10-18

**Authors:** Bamba Gaye, Nzechukwu Michael Isiozor, Gurbinder Singh, Ngone Diaba Gaye, Mame Madjiguene Ka, Daouda Seck, Khadidiatou Gueye, David Lagoro Kitara, Camille Lassale, Anne Malick, Mor Diaw, Sidy M Seck, Abdourahmane Sow, Magaye Gaye, Abdou Salam Fall, Aïda Diongue, Ibrahima Seck, Jamal Belkhadir, Issa Wone, Serigne Magueye Gueye, Papa Salif Sow, Jamal Eddine Kohen, Dorothea Vogelsang, Maïmouna Ndour Mbaye, Elisabeth Alice Liyong, André Pascal Kengne, Roberta Lamptey, Ndeye Marième Sougou, Eugène Sobngwi, Awa Ba, John Tukakira, Thiess Lorenz, Elise G Kabore, Malaizyo Gabriel Muzumala, Ahmed Olanrewaju, Lamin ES Jaiteh, Lucrèce M Delicat-Loembet, Aimée Olivat Rakoto Alson, Khadim Niang, Ciira Wa Maina, Ernest Mwebaze, Joyce Nabende, Dina Machuve, Prestone Adie, Fatoumata Hanne, Roger Tine, Marième Sougou, Kouassi Gustave Koffi, Lameck Luwanda, Elisabeth Lilian Pia Sattler, Demeke Mekonnen, Fatma Ebeid, Jean Paul Enama, Mohamadi Zeba, Fernand Guedou, Pascal Mbelesso, Jennifer Carter, Bakary Coulibaly, Mohamed Lamine Drame, Alain Mouanga, Pierre Marie Preux, Philippe Lacroix, Mouhamadou Diagana, Didier Koumavi Ekouevi, Dismand Houinato, Adama Faye, Vivien Wambugu, Jean Kamaté, Mathias Lalika, Elaine Nsoesie, Boni Maxime Ale, Ibrahima Socé Fall, Abdoulaye Samb, Léon Tshilolo, Modou Jobe

**Affiliations:** 1 Alliance for Medical Research in Africa (AMedRA); 2 Department of Physiology, Cheikh Anta Diop University of Dakar, Dakar, Senegal; 3 Biomedical Informatics, Emory School of Medicine in Atlanta, Georgia, USA; 4 Institute of Clinical Medicine, University of Eastern Finland, Kuopio, Finland; 5 Department of Cardiac Rehabilitation, Ibra Mamadou Wane Medical Center, Dakar, Senegal; 6 Department of Cardiology, Principal Hospital of Dakar, Dakar, Senegal; 7 Institut Pasteur de Dakar, Dakar, Senegal; 8 Fann National University Hospital, Dakar, Senegal; 9 Gulu University, Faculty of Medicine, Department of Surgery, Gulu, Uganda; 10 Barcelona Institute for Global Health (ISGlobal), Barcelona, Spain; 11 Universitat Pompeu Fabra, Barcelona, Spain; 12 CIBER Physiopathology of Obesity and Nutrition (CIBEROBN), Carlos III Health Institute (ISCIII), Madrid, Spain; 13 Ministry of Health and Social Action, Dakar, Senegal; 14 Faculty of Health Sciences, University Gaston Berger, Saint-Louis, Senegal; 15 Surgery & Surgical Specialties Department, Cheikh Anta Diop University of Dakar, Dakar, Senegal; 16 Laboratoire des Transformations Économiques et Sociales, Institut Fondamental d'Afrique Noire Cheikh Anta Diop, Cheikh Anta Diop University, Dakar, Senegal; 17 Senegalese National Agency of Civil Aviation and Meteorology (ANACIM), Dakar, Senegal; 18 Department of Public Health, University Cheikh Anta Diop, Dakar, Senegal; 19 Moroccan League for the Fight Against Diabetes, Rabat, Morocco; 20 Département des Sciences de la Santé, Université Assane Seck de Ziguinchor, Ziguinchor, Sénégal; 21 Department of Infectious Diseases, Fann National University Hospital, Dakar, Senegal; 22 Universite Abulcasis (Cheikh Zaid), Rabat, Morocco; 23 Faculty of Health, Medicine and Life Sciences, Maastricht University, Maastricht, Netherlands; 24 Department of Internal Medicine and Specialties, Cheikh Anta Diop University of Dakar, Dakar, Senegal; 25 Likak Research, Dakar, Senegal; 26 Non-Communicable Diseases Research Unit, South African Medical Research Council, Cape Town, South Africa; 27 Family Medicine Department, Korle Bu Teaching Hospital, Accra, Ghana; 28 Department of Internal Medicine and Specialties, University of Yaounde 1, Yaounde, Cameroon; 29 Laboratoire de physiologie, département de médecine, UFR Santé et Développement Durable, Université Alioune Diop de Bambey, Bambey, Sénégal; 30 Department of Epidemiology, School of Public Health, University of Pittsburgh, Pittsburgh, Pennsylvania, USA; 31 Department of Cardiology, University Heart and Vascular Center Hamburg, University Medical Center Hamburg-Eppendorf, Hamburg, Germany; 32 Department of Cardiology, CHU Yalgado Ouedraogo, Ouagadougou, Burkina Faso; 33 The University of Zambia, Department of Computer Science, University of Zambia Datalab and Enterprise Medical Imaging in Zambia Project, Lusaka, Zambia; 34 University of Ibadan, Ibadan, Nigeria; 35 Department of Surgery, Edward Francis Small Teaching Hospital, Banjul, Gambia; 36 Department of Biology, University of Sciences and Technology of Masuku, Franceville, Haut-Ogooue, Gabon; 37 NGO Sickle Cell Disease Organization of Gabon (SCDOGa), Franceville, Haut-Ogooue, Gabon; 38 Laboratory of Molecular Biology of Joseph Ravoahangy Andrianavalona University Hospital Center (JRA UHC), Faculty of Medicine, University of Antananarivo, Antananarivo, Madagascar; 39 UFR-2S, Université Gaston Berger, Saint-Louis, Senegal; 40 Department of Electrical and Electronic Engineering, Dedan Kimathi University of Technology, Nyeri, Kenya; 41 College of Computing & Information Sciences, Makerere University, Kampala, Uganda; 42 Department of Computer Science, Makerere University, Kampala, Uganda; 43 Schools of Life Sciences and Bioengineering, Nelson Mandela African Institution of Science and Technology, Arusha, Tanzania; 44 ENGIE Energy Access; 45 Institut de Santé et Développement (ISED), Université Cheikh Anta Diop, Dakar, Senegal; 46 Clinical Hematology Unit, CHU de Yopougon, BP 3709 Abidjan 08, Abidjan, Côte d'Ivoire; 47 Haydom Lutheran Hospital in Manyara, Haydom, Tanzania; 48 Department of Nutritional Sciences, University of Georgia, Athens, Georgia, USA; 40 Department of Pediatrics and Child Health, St. Peter Specialized Hospital, Addis Ababa, Ethiopia; 50 Medical Research Lounge Trading PLC, Addis Ababa, Ethiopia; 51 Pediatric Hematology Oncology and BMT, Ain Shams University, Cairo, Egypt; Faculty of Medicine, Ain Shams University Research Institute-Clinical Research Center, Cairo, Egypt; 52 Humanity First, Yaoundé, Cameroon; 53 International Research Laboratory Environnement, Santé Et Sociétés (IRL 3189, ESS), CNRST/CNRS/UCAD/UGB/USTTB, Ouagadougou, Burkina Faso; 54 Laboratoire de Recherche en Maladies Infectieuses Et Parasitaires (LR/MIP), Institut de Recherche en Sciences de La Santé (IRSS/CNRST), Ouagadougou, Burkina Faso; 55 Laboratory of Chronic and Neurologic Diseases Epidemiology, LEMACEN, Doctoral School of Health Sciences, University of Abomey-Calavi, Cotonou, Benin; 56 EMAC-AOC Group (Epidemiology of Chronic Diseases – Central and Western Africa); 57 Department of Neurology, Amitié Hospital, Bangui, Central African Republic; 58 Clinical Trial Service Unit & Epidemiological Studies Unit, Nuffield Department of Population Health, University of Oxford, Oxford, UK; 59 Health Data Research UK, University of Oxford (HDRUK-Oxford), Oxford, UK; 60 Laboratoire de Biologie Moléculaire Appliquée, Bamako, Mali; 61 Health Policy and Health System Expert, International Consultant, Port Moresby, Guinea; 62 Faculté des Sciences et Techniques, Université Marien Ngouabi, Brazzaville, Congo; 63 Inserm U1094, IRD UMR270, Univ. Limoges, CHU Limoges, EpiMaCT – Epidemiology of chronic diseases in tropical zone, Institute of Epidemiology and Tropical Neurology, OmegaHealth, Limoges, France; 64 Département de Neurologie, CHU de Nouakchott, Nouakchott, Mauritania; 65 Faculté des Sciences de la Santé, Département de Santé Publique, Université de Lomé, Lomé, Togo; 66 Duke University, Durham, USA; Duke-Margolis Center for Health Policy, Durham, North Carolina, USA; 67 AGCS PLUS, Advocacy Officer, Dakar, Senegal; 68 Department of Cardiovascular Medicine, Mayo Clinic College of Medicine, Rochester, Minnesota, USA; 69 Department of Global Health, Boston University School of Public Health, Boston, Massachusetts, USA; 70 Institute of Tropical and Infectious Diseases, University of Nairobi, Nairobi, Kenya; 71 Holo Global Health Research Institute, Nairobi, Kenya; 72 School of Public Health, MOI University, Eldoret, Kenya; 73 Department of Health Emergency Interventions, Health Emergencies Programme, World Health Organization, Geneva, Switzerland; 74 Institut de Recherche Biomédicale, CEFA-Monkole, Kinshasa, Democratic Republic of the Congo; 75 Département de Pédiatrie, Université Officielle de Mbujimayi (UOM), Mbujimayi, Democratic Republic of the Congo; 76 Medical Research Council Unit The Gambia at London School of Hygiene & Tropical Medicine, Serrekunda, Gambia

African researchers continue to face numerous barriers that limit the global dissemination and advancement of their research. These include high open-access publication fees, requirements for publishing in high-impact journals, inability to attend or host international conferences, and limited access to published papers and resources. In this viewpoint, we focus on the specific challenge of attending international scientific conferences hosted in high-income settings and aim to propose practical solutions to address this inequity. The related barriers include visa-related difficulties and prohibitive travel expenses (e.g. non-reimbursable high visa fees, flight tickets, accommodation and subsistence, and conference registration costs).

## The ‘indirect’ rationale justifying the need to have African researchers ‘at the table’

The coronavirus disease 2019 (COVID-19) pandemic has undoubtedly underscored the need for international collaborations among researchers worldwide, particularly in the health sector. Such collaborations are essential for combining resources, sharing expertise, and effectively addressing global health challenges through collective knowledge and coordinated efforts in a time-efficient manner. International conferences are key in this process, offering a platform for researchers from diverse backgrounds to exchange ideas, share findings, and collectively develop strategies for tackling challenging health issues.

## The overall visa landscape

Despite relatively higher disease burden in several low- and middle-income countries (LMICs) compared to high-income countries (HICs) [1], high-profile conferences, such as the World Health Summit, the International Society of Global Health Conference, and the International AIDS Society (IAS) Conference, are usually hosted in high-income countries. These HIC host nations commonly have mutual visa waiver agreements with other HICs, but such agreements are rarely extended to LMICs, including African nations (**Table 1**) [2−5]. This situation leaves researchers from LMICs facing the significant challenge of securing visas for travel.

**Table 1:** Visa-waiver countries for the USA, UK, and the Schengen region

USA [2]	UK [3,4]	Schengen [5]
Andorra, Australia, Austria, Belgium, Brunei, Chile, Croatia, Czech Republic, Denmark, Estonia, Finland, France, Germany, Greece, Hungary, Iceland, Ireland, Italy, Japan, Latvia, Liechtenstein, Lithuania, Luxembourg, Malta, Monaco, Netherlands, New Zealand, Norway, Poland, Portugal, San Marino, Singapore, Slovakia, Slovenia, South Korea, Spain, Sweden, Switzerland, Taiwan, United Kingdom	Australia, EU countries, USA, Iceland, Canada, Japan, Liechtenstein, New Zealand, Norway, Norway, Singapore, South Korea, Switzerland, Kuwait, Oman, Qatar, United Arab Emirates*	Albania, Antigua and Barbuda, Argentina, Australia, Bahamas, Barbados, Bosnia and Herzegovina, Brazil, Brunei, Canada, Chile, Colombia, Costa Rica, Dominica, El Salvador, Georgia, Grenada, Guatemala, Honduras, Hong Kong, Israel, Japan, Kiribati, Macao, North Macedonia, Malaysia, Marshall Islands, Mauritius, Mexico, Micronesia, Moldova, Montenegro, New Zealand, Nicaragua, Palau, Panama, Paraguay, Peru, Saint Kitts and Nevis, Saint Lucia, Saint Vincent, Samoa, Serbia, Seychelles, Singapore, Solomon Islands, South Korea, Taiwan, Timor Leste, Tonga, Trinidad and Tobago, Tuvalu, Ukraine, United Arab Emirates, United Kingdom, United States of America, Uruguay, Vanuatu, Venezuela†

*Kuwait, Oman, Qatar, and the United Arab Emirates had a waiver of GBP 30.

†Seychelles and Mauritius are the only African countries in the programme.

An overview of visa-waiver programmes for top health conference destinations – USA, UK, Canada, and the Schengen region (including European countries such as France, Germany, and Spain) – shows that no African country is included in the visa-waiver programmes for the USA and UK. Notably, only two African countries, Mauritius and Seychelles, are part of the Schengen visa-waiver programme [6].

## Detailing visa-related challenges among African researchers

Most African researchers, whether conducting research within the African continent or abroad, face significant challenges in physically attending these scientific gatherings. These barriers include the stress and uncertainties associated with obtaining visas, in addition to financial challenges. The visa application process, often with a processing period of several weeks or months or even up to a year, is lengthy, preventing African researchers from making timely decisions to attend international conferences. Consequently, their participation in these scientific events largely depends on the visa-issuing authorities of the host countries. According to a previous study, African researchers are over three times more likely to encounter visa-related obstacles compared to their North American and European counterparts [7]. This issue was observed during the 2022 IAS conference, where numerous visa delays and denials by Canadian authorities prevented many registered delegates, including IAS staff, from participating [8]. A survey by the RAND Corporation for the Wellcome Trust identified the duration of visa requests, complexity of application forms, and application costs as the primary complaints among African and Asian researchers [7].

## The negative impact of visa-related barriers

A systematic review has reported the underrepresentation of participants from LMICs, which include all 54 African countries, in global health conferences [9]. Persistent visa-related challenges are likely to worsen this underrepresentation, resulting in missed opportunities for African researchers to enhance their knowledge, present their research on a global platform, and establish valuable networking connections with other international experts. In contrast, researchers from countries included in visa-waiver programmes offered by host nations experience fewer barriers to attendance and thus benefit more from these opportunities. Consequently, their participation rates are much higher, leading to greater knowledge exchange, more networking opportunities, and access to the latest information on current health issues. This disparity in participation and opportunity has been referred to as ‘conference inequity’ [9].

The burden of both communicable and non-communicable diseases (NCDs) in Africa remains a major challenge. NCDs are responsible for 41 million deaths annually, accounting for 74% of all deaths worldwide. Of those, 17 million people die from NCDs before reaching the age of 70, with 86% of these premature deaths occurring in LMICs. In fact, 77% of all NCD-related deaths happen in these regions [10]. The growing prevalence of chronic diseases, coupled with the existing burden of infectious diseases in the African continent, underscores the necessity for African researchers to enhance their skills and integrate into established global networks. This integration would enable the African scientific community to present its research findings and interventional approaches for improving health at the international level.

However, several challenges must be addressed to achieve this, including visa application protocols and associated uncertainties, the rising visa fees, flight ticket costs, and registration fees, all of which are further complicated by fluctuating local exchange rates. Among these challenges, visa processing remains the most critical issue.

## Broadening the scope beyond conference attendance: Addressing global barriers to mobility and collaboration

To address the broader issue beyond just the challenges of attending international scientific conferences, it is essential to consider the full scope of barriers that African researchers face when attempting to collaborate globally. The constraints extend well beyond the logistics of conference attendance and include significant obstacles to international travel, research collaboration, and access to global research networks. These barriers are not just administrative or financial, but are deeply rooted in systemic inequities that disproportionately affect researchers from LMICs. The issue is not just about missing out on conferences but also about missing out on critical opportunities for professional growth, knowledge exchange, and contribution to global scientific discourse.

Moreover, the isolation caused by these barriers delays the progress of African research, limiting the continent’s ability to influence global health strategies and contribute solutions that are contextually relevant to the African experience. The global research community thereby loses out on the unique insights that African researchers provide, especially in fields where Africa bears a disproportionate share of the global burden and has developed extensive expertise, such as infectious diseases and emerging NCDs.

To truly bridge this gap, efforts must focus on creating more inclusive and accessible pathways for African researchers to travel, collaborate, and contribute on a global scale. This includes advocating for more equitable visa policies, securing funding for travel and collaboration, and fostering an international research environment that recognises and actively mitigates these barriers. Addressing the broader issue of mobility and collaboration will ensure that African researchers are fully integrated into the global research ecosystem, enriching it with their perspectives and expertise.

## The issue of citizenship and brain drain

A persistent and often overlooked consequence of the stringent visa and mobility barriers faced by African researchers is the forced prolongation of their stay in Western countries to secure citizenship. This situation arises because, after completing their studies or research training abroad, many African citizens find themselves in a vulnerable position. Returning to their home countries often means giving up the opportunity to apply for permanent residency or citizenship in the host country, which would facilitate easier future travels and career opportunities.

Consequently, numerous African researchers opt to remain in Western countries for several years after their formal training, navigating the lengthy and complex process of obtaining permanent residency or citizenship. This extended stay is not merely a personal choice, but rather a strategic decision to avoid the recurring visa-related challenges that significantly limit their ability to participate in global scientific engagements.

However, this decision comes at a substantial cost to the African continent. The delayed return of highly skilled professionals intensifies the already critical issue of brain drain, wherein talented and educated individuals are lost to nations that offer more favourable conditions for professional growth and mobility. The resultant effect is a weakening of the research and development capabilities within African nations, which are in urgent need of these skilled individuals to address pressing local and regional health challenges. Moreover, the prolonged absence of these researchers diminishes their immediate contribution to the academic and scientific landscape in Africa, creating a gap in mentorship for the next generation of African scientists. It also delays the potential for establishing robust, locally-led research initiatives that are crucial for Africa’s sustainable development.

Addressing this issue requires a multi-faceted approach. Host countries should consider policy reforms that facilitate the return of African researchers by granting them the flexibility to engage in international research collaborations without the fear of losing residency rights. Additionally, African governments and institutions should provide stronger incentives and support systems to encourage the return of their diaspora, ensuring that the skills and knowledge gained abroad are reinvested into the continent's development.

Ultimately, the goal should be to create an environment where African researchers can thrive both at home and abroad, without being forced into difficult decisions that contribute to the brain drain phenomenon. This requires global recognition of the unique challenges they face and concerted efforts to remove the barriers that drive them away from their home countries.

## References

[1] van der Ham M Bolijn R de Vries A Campos Ponce M van Valkengoed IGM Gender inequality and the double burden of disease in low-income and middle-income countries: an ecological study. BMJ Open. 2021;11:e047388. 10.1136/bmjopen-2020-047388 33895719 PMC8074552

[2] U.S. Department of State. Visa Waiver Program. 2021. Available: https://travel.state.gov/content/travel/en/us-visas/tourism-visit/visa-waiver-program.html#reference. Accessed: 3 October 2024.

[3] Government of the United Kingdom. UK visa requirements (accessible version). 2024. Available: https://www.gov.uk/government/publications/uk-visa-requirements-list-for-carriers/uk-visa-requirements-for-international-carriers. Accessed: 3 October 2024.

[4] Government of the United Kingdom. Electronic visa waivers.2024. Available: https://www.gov.uk/get-electronic-visa-waiver#:~:text=You%20can%20get%20an%20electronic,business%2C%20study%20or%20medical%20treatment. Accessed: 3 October 2024.

[5] European Travel Information and Authorisation System. ETIAS Countries. 2024. Available: https://etias.com/etias-countries/. Accessed: 3 October 2024.

[6] Schengen Visa Info. List of visa-exempt nationalities. 2024. Available: https://schengenvisum.info/en/what-is-schengen-visa/schengen-countries/list-of-nationalities-not-requiring-a-visa/. Accessed: 3 October 2024.

[7] Nature. African and Asian researchers are hampered by visa problems. 2018. Available: https://www.nature.com/articles/d41586-018-06750-1. Accessed: 3 October 2024.

[8] Medscape. Organizers Blame 'Systemic Racism' for AIDS Conference Visa Fiasco. 2022. Available: https://www.medscape.com/viewarticle/979394. Accessed: 3 October 2024.

[9] Velin L Lartigue JW Johnson SA Zorigtbaatar A Kanmounye US Truche P Conference equity in global health: a systematic review of factors impacting LMIC representation at global health conferences. BMJ Glob Health. 2021;6:e003455. 10.1136/bmjgh-2020-003455 33472838 PMC7818815

[10] World Health Organization. Noncommunicable diseases. 2023. Available: https://www.who.int/news-room/fact-sheets/detail/noncommunicable-diseases. Accessed: 3 October 2024.

